# Novel African Trypanocidal Agents: Membrane Rigidifying Peptides

**DOI:** 10.1371/journal.pone.0044384

**Published:** 2012-09-07

**Authors:** John M. Harrington, Chris Scelsi, Andreas Hartel, Nicola G. Jones, Markus Engstler, Paul Capewell, Annette MacLeod, Stephen Hajduk

**Affiliations:** 1 Department of Biochemistry and Molecular Biology, University of Georgia, Athens, Georgia, United States of America; 2 Department of Cell and Developmental Biology, Theodor-Boveri-Institute, University of Wuerzburg, Wuerzburg, Germany; 3 Wellcome Trust Centre for Molecular Parasitology, College of Medical, Veterinary and Life Sciences, University of Glasgow, Glasgow, United Kingdom; INSERM U1094, University of Limoges School of Medicine, France

## Abstract

The bloodstream developmental forms of pathogenic African trypanosomes are uniquely susceptible to killing by small hydrophobic peptides. Trypanocidal activity is conferred by peptide hydrophobicity and charge distribution and results from increased rigidity of the plasma membrane. Structural analysis of lipid-associated peptide suggests a mechanism of phospholipid clamping in which an internal hydrophobic bulge anchors the peptide in the membrane and positively charged moieties at the termini coordinate phosphates of the polar lipid headgroups. This mechanism reveals a necessary phenotype in bloodstream form African trypanosomes, high membrane fluidity, and we suggest that targeting the plasma membrane lipid bilayer as a whole may be a novel strategy for the development of new pharmaceutical agents. Additionally, the peptides we have described may be valuable tools for probing the biosynthetic machinery responsible for the unique composition and characteristics of African trypanosome plasma membranes.

## Introduction

Eukaryotic pathogens of the genus *Trypanosoma* cause deadly disease in humans and livestock. Human African trypanosomiasis, or sleeping sickness, is caused by two subspecies, *Trypanosoma brucei rhodesiense*, causing an acute disease, or *Trypanosoma brucei gambiense*, causing a chronic infection [1]. Nagana, an economically debilitating wasting disease in African cattle is caused by *Trypanosoma brucei brucei* and other species including *Trypanosoma vivax* and *Trypanosoma congolense* [2]. *Trypanosoma vivax* infection in cattle has also been established in South America [3]. African trypanosomes exhibit several life stages including a bloodstream form (BSF) in the circulation of the mammalian host, a procyclic form (PCF) in the midgut of the tsetse fly vector and a metacyclic form that is injected during fly-feeding and initiates infection [4].

Drug development is difficult for a number of reasons, both biological and economic, and resistance is also a major problem [5]. Higher primates are innately immune to veterinary pathogenic African trypanosomes due to circulating trypanolytic factors [6], [7]. However African trypanosomes have evolved a variety of mechanisms to evade innate and acquired immunity. The best-understood mechanism is successive expression of antigenically distinct glycerophosphotidylinositol-anchored (GPI-) variant surface glycoproteins (VSG) [8]. Another strategy is clearance of surface bound host defense factors. The most striking example of this is the hydrodynamic flow-mediated lateral sorting of Ig-bound-VSG to the flagellar pocket, an invagination at the posterior of the cell that is the sole site for endocytosis [9].

Previously we demonstrated that both veterinary and human pathogenic BSF *T. brucei* are uniquely susceptible to killing by two small hydrophobic peptides (SHP), SHP-1 and -2 [10]. Specificity of SHP is mediated by a high degree of fluidity in the plasma membrane of BSF cells [10]. These peptides do not bind, and thus do not kill, PCF *T. brucei*, which has a more rigid plasma membrane. Human cells, including erythrocytes, are refractory to SHP concentrations orders of magnitude higher than necessary to kill BSF *T. brucei* [10].

Here we report that trypanocidal SHP uniquely cause an increase in the rigidity of the interfacial region of the plasma membrane that is consistent with dramatic motility constriction and subsequent cell death. We present an explanation, based upon sequence analysis and the orientation and structure of lipid associated SHP, for these biophysical consequences.

## Results

### Susceptibility to SHP is Independent of VSG and Common to African Trypanosomes

**Figure 1:**
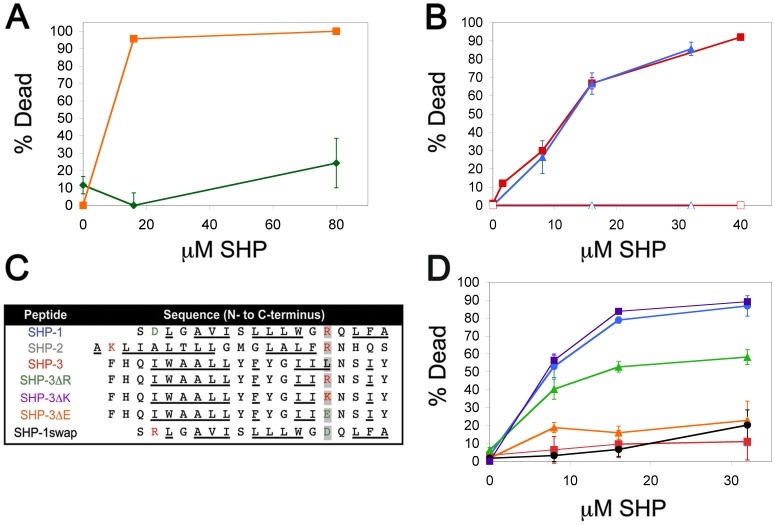
Small hydrophobic peptide mediated killing of African trypanosomes. (**a**) The metacyclic developmental form (the developmental stage injected during a tsetse fly bite) of *T. b. brucei* was assayed for susceptibility to SHP-1 in 2 h *in vitro* killing assays (green ⧫, metacyclic; orange ▪, BSF). (**b**) The veterinary pathogenic African trypanosomes, *T. vivax* (red ▪, SHP-1; red-open □, DMSO) and *T. congolense* (blue ▴, SHP-1; blue-open ▵, DMSO), were assayed for susceptibility to SHP-1 in 2 h *in vitro* killing assays. (**c**) The sequences of trypanolytic and non-trypanolytic SHP are shown from N- to C-terminus and aligned to the C-terminus in order to emphasize the identity of the amino acid at position -5 relative to the putative signal peptidase cleavage site. Positively charged amino acids are in red, negatively charged amino acids are in green and non-polar amino acids are underlined. (**d**) The SHP listed in (c) were tested against BSF *T. b. brucei* in 2 h killing assays (blue •, SHP-1; red ▪, SHP-3; green ▴, SHP-3ΔR; purple ▪, SHP-3ΔK; orange ▴, SHP-3ΔE; black •, SHP-1swap). Colors correspond to the peptide names in (c).

An immediately apparent difference between the plasma membranes of BSF and PCF African trypanosomes is the lack of a dense coat of VSG in the insect stage cells. We tested metacyclic *T. b. brucei*, which do express a VSG coat, for susceptibility to SHP-1 and found no killing activity (Fig. 1a). These data indicate that susceptibility is not due to specificity of SHP for VSG. Next we determined whether other African trypanosomes are sensitive to SHP. Bloodstream developmental forms of *T. vivax* and *T. congolense* are susceptible to killing by SHP-1 at concentrations similar to BSF *T. brucei* (Fig. 1b), indicating that SHP susceptibility is a characteristic of both human and veterinary pathogenic African trypanosomes.

### Trypanocidal Activity Requires a C-terminal Positive Charge

Trypanocidal SHP are derived from apolipoproteins and exhibit the characteristics of secretory signal peptides, i.e. size (18–22 amino acids), a central hydrophobic region and a C-terminal putative signal peptidase cleavage site defined by specific amino acid patterns [10]. Although these peptides share physical features, the primary sequences are entirely different (Fig. 1c). We tested a third, distinct SHP (−3) for trypanocidal activity, also derived from an apolipoprotein [11] and possessing similar features as SHP-1 and -2 (Fig. 1c). Despite possessing the same general physical characteristics and binding to BSF *T. brucei* (Methods File S1, Figure S1), no trypanocidal activity was detected (Fig. 1d). Comparison of the three sequences revealed that an arginine at position -5 relative to the C-terminus is common to trypanocidal SHP-1 and -2, but is absent in SHP-3 (Fig. 1c). Substitution of an arginine for the leucine in this position of SHP-3 (SHP-3ΔR, Fig. 1c) confers trypanocidal activity (Fig. 1d). Replacement of the leucine with glutamate in SHP-3 (SHP-3ΔE, Fig. 1c) does not (Fig. 1d). Trypanocidal activity is conferred simply by a positive charge at the C-terminus, indicated by the trypanocidal activity of an SHP-3 variant in which the -5 leucine is replaced by lysine (SHP-3ΔK, Fig. 1d). Trypanocidal SHP-2 has a positive charge at both the N- and C-terminus, SHP-1 has a single positive charge at the C-terminus; thus we tested whether charge location is important by swapping the C-terminal arginine with the N-terminal aspartate of SHP-1 (SHP-1-swap, Fig. 1c). Rearranging these residues resulted in a loss of trypanocidal activity (Fig. 1d).

### Trypanocidal SHP Rigidify the Interior and Interfacial Region of the Plasma Membrane

**Figure 2:**
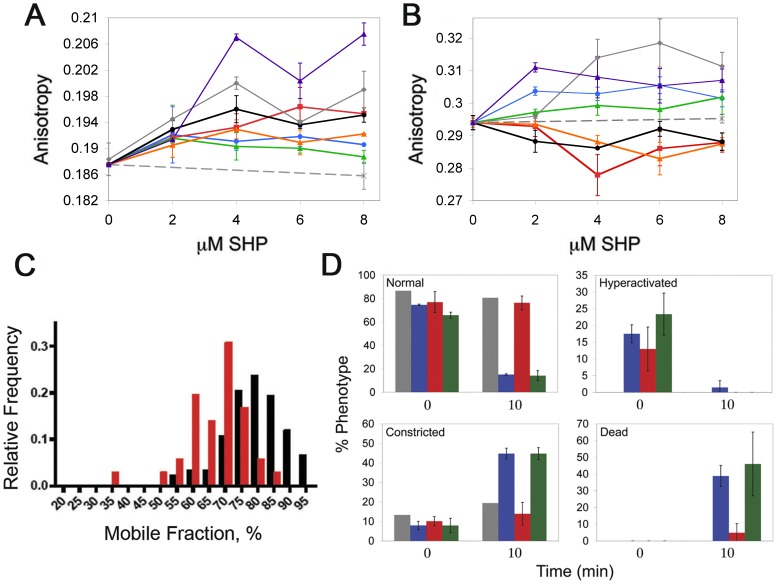
Membrane rigidity changes and physiological consequences of SHP. The rigidity of the interior (**a**) or interfacial (**b**) region of the plasma membrane of BSF *T. b. brucei* treated with increasing concentrations of SHP-1 (blue •), SHP-2 (grey ⧫), SHP-3 (red ▪), SHP-3ΔR (green ▴), SHP-3ΔK (purple ▴), SHP-3ΔE (orange-open ▵), SHP-1swap (black-open ○) or solvent alone (DMSO, black ×) was determined by measuring the fluorescence depolarization of DPH or TMA-DPH respectively. (**c**) FRAP analysis of the mobile fraction of BSF *T. b. brucei* VSG in the presence (red ▪) or absence (black ▪) of 8 µM SHP-1. (**d**) Live BSF *T. b. brucei* treated with equivolume DMSO (grey), 40 µM SHP-1 (blue), SHP-3 (red) or SHP-3ΔR (green) were visualized by DIC microscopy and scored for normal, hyperactivated and constricted motility as well as death at the indicated timepoints (see Movies S1, S2, S3 and S4 for examples of the normal, hyperactivated and constricted motilities respectively).

Trypanocidal SHP act at the plasma membrane but do not induce osmotic swelling or bursting [10]. Therefore we reasoned that any effect upon the BSF trypanosome must not result in a loss of plasma membrane integrity. We investigated the rigidity of BSF *T. brucei* membranes, a property that can change without loss of membrane integrity, utilizing two anisotropic probes, diphenylhexatriene (DPH) that reports on the interior of the acyl chain region, and trimethylammonium-diphenylhexatriene (TMA-DPH) that is anchored at the membrane interface. Addition of either trypanocidal or non-trypanocidal SHP to BSF *T. brucei* results in increased rigidity of the interior of the plasma membrane (Fig. 2a). However only the trypanocidal SHP, SHP-1, -2, -3ΔR and -3ΔK increased the interfacial rigidity (Fig. 2b). These data indicate that rigidification of the interfacial region is likely involved in killing BSF trypanosomes.

### Trypanocidal SHP have Biophysical and Physiological Consequences

Treatment of BSF African trypanosomes with SHP results in multiple physiological alterations. Addition of SHP-1 to BSF trypanosomes, which were subsequently immobilized in gelatin, decreases the fraction of laterally mobile surface exposed VSG (Fig. 2c). This effect may be directly due to increased membrane rigidity, or indirectly due to a decrease in the mobility of membrane spanning proteins and potential interactions with the VSG. Another physiological consequence, that may or may not be related to membrane rigidification, is SHP-induced changes in cell motility. Previously we reported that SHP-1 causes an initial hyperactivation followed by constricted motility and death [10]. Non-trypanocidal SHP-3 does cause some hyperactivation of BSF *T. b. brucei* (Movie S1 displays an untreated cell for comparison, Movie S2 presents a representative cell exhibiting SHP-3 induced hyperactivation), however subsequent constriction does not occur (Fig. 2d). Trypanocidal SHP-1 [10] and SHP-3ΔR (Movie S3, S4) induce both hyperactivation and subsequent constriction (Fig. 2d). Constricted motility may result in reduced hydrodynamic forces acting upon surface proteins.

### Trypanocidal SHP Exhibit Shallow Penetration and Orient Perpendicular to the Plane of the Membrane

**Figure 3:**
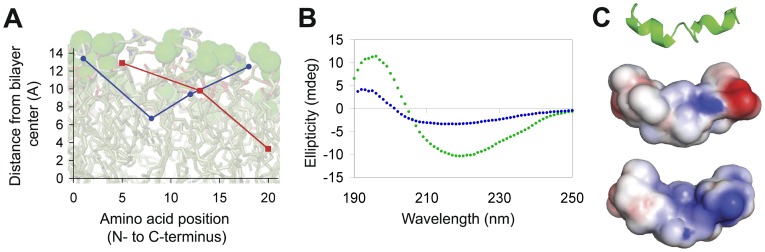
Orientation and structure of SHP in lipid bilayers. (**a**) The depth of peptide penetration into the hydrocarbon region of model liposomes was determined via parallax analysis. Assuming a hydrocarbon bilayer thickness of 29 Å, the depths of tryptophans spanning SHP-1 (blue •) and SHP-3 (red ▪) are plotted against a background of the outer leaflet of a POPC bilayer. (**b**) Circular dichroism spectroscopy of SHP-1 in aqueous buffer (green •) and in the presence of egg phosphatidylcholine liposomes (blue •). (**c**) Molecular dynamic modeling of SHP-1 in a lipid environment. The backbone trace (top) illustrates a predominantly α-helical structure with disordered termini and an internal disordered region. Surface potential representations (N to C terminal, middle; C to N terminal, bottom) indicate positively charged patches (blue) formed by the N-terminal amino acid and the arginine at position 14. Non-polar and negatively charged regions are shown in white and red, respectively.

In order to understand why trypanocidal and non-trypanocidal SHP have different effects on the BSF plasma membrane, we determined the orientation of SHP-1 and -3 in lipid bilayers by parallax analysis [12]. Tryptophans were substituted at positions 1, 8 and 18 (N- to C-terminus, native tryptophan located at position 12) in SHP-1 and positions 1, 13 and 20 in SHP-3 (N- to C- terminus, native tryptophan located at position 5) (Table S1). These placements were chosen, and native tryptophan residues were replaced with glycine, in order to retain the hydrophobic profile of the original peptides. All of the substituted SHP-1 peptides show equivalent killing activity as well as membrane interaction (Methods File S1, Figure S2a, b). We determined the insertion depth of SHP tryptophans by ratiometric analysis of the quenching efficiency of liposomes containing 10 mol % brominated lipid at a shallow (6,7) and deep (9,10) position of the acyl chains. Trypanocidal SHP-1 penetrates shallowly into the hydrocarbon region and adopts a U-shaped conformation (Fig. 3a, Table S1). The two terminal tryptophans, positions 1 and 18, are located approximately 1.1 and 2.0 Å from the membrane interface respectively. The tryptophans at positions 8 and 12 are located approximately 7.8 and 5.1 Å from the interface respectively. Therefore, rather than aligning with the phospholipid acyl chains, SHP-1 inserts into the exterior leaflet parallel to the plane of the bilayer and proximal to the phospholipid headgroups. These data are consistent with an orientation that has been observed for the LamB signal peptide [13], [14]. Non-trypanocidal SHP-3 and the tryptophan variants also exhibit membrane interaction (Figure S2c). Parallax analysis of SHP-3 indicates a tilted orientation with the C-terminus penetrating deeper into the bilayer, an orientation that has also been suggested for the LamB signal peptide (Fig. 3a, Table S1) [13]. The N-terminal tryptophan was inefficiently quenched, suggesting that it does not intercalate into the hydrocarbon region. The native tryptophan, position 5, inserts approximately 1.6 Å deep. The tryptophan at position 13 is located approximately 4.7 Å deep and the C-terminal tryptophan penetrates most deeply, to approximately 11.2 Å. This orientation precludes interaction of the C-terminus with the lipid headgroups.

### Trypanocidal SHP Adopt α-helical Structures with Positively Charged Patches at Both Termini

Circular dichroism spectroscopy reveals different structures for soluble and lipid-associated SHP-1. In the solution spectrum, the peak at 195 nm, crossover at approximately 205 nm and minimum at 218 nm are characteristic of a β-sheet conformation (Fig. 3b) [15]. In the presence of lipid, the peak at 193 nm and minimum at 208 nm suggest that SHP-1 adopts an α-helical structure (Fig. 3b) [15], again consistent with the secondary structure of the LamB signal peptide [16]. The structural analysis and parallax data are consistent with a predicted structure from molecular dynamics simulations, in which SHP-1 adopts a helix-break-helix, a motif reported for lipid associated PhoE signal peptide [17]. The free amino-group at the N-terminus and the C-terminal arginine form positively charged patches at both termini and the interior region forms a hydrophobic bulge (Fig. 3c).

## Discussion

Changes in membrane rigidity as measured by fluorescence anisotropy have been shown to correlate with alterations in cellular physiology and biochemistry. Temperature induced rigidification of platelet membranes results in a concomitant decrease in fluid phase endocytosis [18]. Additionally in the type 1 diabetic disease state platelets exhibit an increase in the rigidity of the interior of the plasma membrane that results in a decrease in Na^+^/K^+^-ATPase activity [19]. In the case of BSF African trypanosomes, we have correlated increased membrane rigidity with changes in cell motility, the diffusion of rate of surface proteins and ultimately cell death. Therefore, membrane rigidification may be trypanocidal by interfering with a variety of activities such as, but not limited to, lateral surface protein diffusion, ion channel function and/or the diffusion of small molecule nutrients into the BSF cell. It has been shown that increasing the rigidity of trypanosome membranes results in a redistribution of proteins normally localized to the flagellar membrane [20], posing the possibility that signaling pathways may be affected. Therefore trypanocidal SHP may have pleiotropic effects.

In addition to directly killing BSF trypanosomes, SHP induce physiological changes that may attenuate the parasites’ ability to evade host immune effectors. The diffusion of VSG is altered in trypanosomes treated with SHP-1. The decrease in the number of mobile surface VSG may be due to increased membrane rigidity conferring greater drag upon the myristate anchors. Alternatively, or in addition to, increased membrane rigidity may hinder VSG diffusion through decreasing the mobility of membrane spanning proteins that subsequently interfere with the lateral surface flow of VSG. In either case hindering the flow of VSG to the flagellar pocket would likely delay the clearance of host defense molecules thereby promoting cell killing. Additionally, constricting the motility of BSF trypanosomes may effectively decrease hydrodynamic flow over the surface of the cell, a force necessary for maintaining directionality and movement of VSG to the anterior of the trypanosome [9], also delaying the clearance of VSG. Employing agents that increase the rigidity of the plasma membrane in pharmaceutical applications may therefore augment the host immune response to trypanosome infection.

The orientation of trypanocidal SHP in lipid bilayers differs from non- trypanocidal SHP in that both termini are proximal to the membrane interface. This orientation can be attributed to a positively charged residue at the -5 position relative to the C-terminus that is lacking in non- trypanocidal SHP. Swapping a negatively charged residue for the C-terminal arginine (SHP-1swap) will result in a similar hydrophobic profile and thus presumably induce the same orientation in lipid bilayers, however this change renders the SHP incapable of rigidifying the membrane interface and therefore non- trypanocidal. These data indicate a direct role for a C-terminal positively charged residue (arginine or lysine) in increasing interfacial rigidity and trypanosome killing. Incorporating the requirement for a positive charge at the C-terminus and the hydrophobic bulge revealed by molecular dynamic modeling, we suggest a mechanism in which trypanocidal SHP are anchored in the membrane hydrocarbon region by the internal hydrophobic stretch and the positively charged patches at each termini coordinate negatively charged phosphates of the lipid headgroups. This model provides a plausible explanation for the increase in interfacial membrane rigidity by trypanocidal but not non- trypanocidal SHP.

The specificity of SHP for BSF African trypanosomes reveals a phenotype that may be taken advantage of for the development of pharmaceutical agents. Drugs that target the fluidity of the plasma membrane may offer a means of circumventing the rapid onset of resistance exhibited by these pathogens. Compensating for a phenotype that is the result of a system of gene products would require multiple viable mutations rather than a single mutation within a targeted enzyme or transporter. Additionally SHP may be valuable tools to investigate the molecular basis of membrane fluidity.

## Materials and Methods

### Peptides and Lipids

All peptides were purchased from Bio-Synthesis, Inc. (Lewisville, TX). All lipids were purchased from Avanti Polar Lipids (Alabaster, AL). These include phosphatidylcholine from egg (8450051) and 1-palmitoyl-2-(6,7-dibromo)stearoyl-sn-glycero-3-phosphocholine (850480) and 1-palmitoyl-2-(9,10-dibromo)stearoyl-sn-glycero-3-phosphocholine (850481).

### Trypanosome Killing Assays

Light microscopy based trypanosome killing assays were performed as previously described in detail [6], [10], [21], [22]. Metacyclic *Trypanosoma brucei brucei* strain STIB247 were obtained from dissection of the salivary glands of infected tsetse flies. Newly hatched *Glossina morsitans morsitans* (24–48 h post eclosion) were fed two defibrinated horse blood meals containing 2×10^6^ mL^-1^ trypanosomes on two separate days. Flies were then maintained at 25°C in 75 % relative humidity and fed maintenance blood meals three times per week. Following day 20–30, flies were harvested and salivary glands were dissected out and placed into HMI 9 media containing 10 % fetal bovine serum for 30 minutes. Metacyclic cells were expelled into the media by the residual peristalsis of the salivary glands. Cells were collected via centrifugation and maintained in HMI 9 media with 20 % fetal bovine serum at 37°C in 5 % CO_2_ until use. Killing assays were conducted with 1×10^4^ cells/mL in HMI 9 media containing 10 % fetal bovine serum. Live cells were scored via hemocytometer after 2 h incubation. *Trypanosoma vivax* strain ILRAD V34 and *Trypanosoma congolense* strain IL3000 were grown from stabilites in donor ICR mouse (Harlan, United Kingdom). Parasites were harvested from mice by terminal exsanguination and subsequent differential centrifugation of the blood with an equal volume of HMI 9 media to form a buffy coat layer. Cells were maintained in HMI 9 with 20 % fetal bovine serum at 37°C in 5 % CO_2_ until use. Killing assays were performed with 1×10^7^ cells/mL in HMI 9 media with 20 % bovine serum. Cells were incubated at 37°C for 2 h and live cells were scored visually via hemocytometer. All assays were conducted in at least duplicate, and data points are the averages with standard deviations.

### Anisotropy Assays

The plasma membrane rigidity of live *T. b. brucei* was determined by measuring the fluorescence depolarization of diphenyl-1,3,5- hexatriene p-toluenesulfonate (DPH) or 1-(4-trimethylammoniumphenyl)-6-diphenyl-1,3,5- hexatriene p-toluenesulfonate (TMA-DPH; Invitrogen T204). Cells were washed 3 times with and resuspended in phosphate buffered saline at a density of 3×10^6^ cells/mL. The anisotropic probes were added to a final concentration of 0.5 µM and allowed to intercalate into the cell membrane for 1 h in the dark. Anisotropic values were acquired via the software function of a PerkinElmer Life Sciences LS55 spectrofluorometer. Samples were excited at 358 nm, and emission was read at 430 nm, with 10-nm excitation and emission slit widths. Temperature was maintained at 37°C by means of the PerkinElmer LS55 Biokinetics accessory. Data were corrected for light scattering with an unlabeled sample of cells, and anisotropy was calculated according to the equation r  =  (I_VV_ - GI_VH_)/(I_VV_ + 2GI_VH_), where r is the anisotropy value, I_VV_ is the emission intensity acquired with the excitation- and emission-polarizing filters set vertically, G is the instrument correction factor, and I_VH_ is the emission intensity acquired with the excitation-polarizing filter set vertically and the emission-polarizing filter set horizontally. Data points shown are the average of triplicate measurements with standard deviations.

### In-vivo Fluorescence Recovery after Photo Bleaching (FRAP) Measurements

Bloodstream form *T. b. brucei*, strain 427, expressing VSG MITat1.6 were cultivated in HMI 9 media with 10% fetal calf serum. 1×10^7^ cells were washed three times with ice cold trypanosome dilution buffer (TDB; 5 mM KCl, 80 mM NaCl, 1 mM MgSO_4_, 20 mM Na_2_HPO_4,_ 2 mM NaH_2_PO_4_, 20 mM glucose, pH 7.6). The cell density was adjusted to 1×10^8^ cells/mL and labeling of surface proteins was achieved by incubation with 1 µM sulfo-NHS coupled Atto 488 fluorescent dye (ATTO-TEC GmbH, Siegen) for 15 min on ice [23]. After incubation the cells were washed three times with ice cold TDB to remove unbound dye. Labeled *T. b. brucei* cells were incubated with 8 µM SHP-1 for 10 min at 37°C. The fluorescently labeled cells were mixed 1∶3 with 10% Type-A gelatin from porcine skin (Sigma-Aldrich, Steinheim) in PBS, pH 7.8, at 37°C. 4 µL of the cell gelatin mixture was applied into a cover slide sandwich and mounted into a temperature controlled sample holder. The sample holder was cooled to 20°C until the cells were immobilized. Samples of SHP-1 treated and untreated MITat 1.6 wt cells were prepared identically. Line FRAP measurements were performed at a constant temperature of 20°C. 10 pre-bleach and 100 post-bleach images were recorded at 2fps. VSG mobile fractions were determined according to Phair et al. (2004) [24] using double normalization. The mobile fraction refers to the percentage of mobile VSG molecules within the measured region. For example a mobile fraction of 50 % means that half of the VSG molecules are mobile. The relative frequency indicates the proportion of cells that exhibit a given mobile fraction of VSG.

### Trypanosome Motility

All images and videos were acquired with an Axio Observer Z1 equipped with an AxioCam MRm controlled by AxioVision 4.6 software. Videos were acquired with live cells at a density of 1×10^7^ cells/mL in HMI 9 media with 10 % fetal bovine serum, incubated with 40 µM SHP at 37°C. Trypanosomes were visualized at magnification 63 × and videos were recorded with 100-ms acquisition times. The motility of BSF trypanosomes was scored directly or from video playback. Trypanosome motility was classified as normal, hyperactive or constricted as described previously [10]. An example of a trypanosome scored as normal is shown in Movie S1. Example hyperactive trypanosomes are shown in Movies S2 and S3, while those exhibiting constricted motility are exemplified by the representative trypanosome presented in Movie S4. Data in figure 2D is shown as the average of at least duplicate trials with standard deviations.

### Parallax Analysis

The hydrocarbon penetration depth of tryptophans spaced throughout synthetic peptides corresponding to SHP-1 or SHP-3 (Table S1) was determined by parallax analysis with brominated phosphatidylcholine liposomes. Large unilamellar liposomes composed of egg phosphatidylcholine and 10 mol % 1-palmitoyl-2-(6,7-dibromo)stearoyl-sn-glycero-3-phosphocholine (shallow quencher) or 1-palmitoyl-2-(9,10-dibromo)stearoyl-sn-glycero-3-phosphocholine (deep quencher) were constructed by hydration of a thin dry lipid film with phosphate buffered saline. Resulting multilamellar liposomes were made unilameller via extrusion through polycarbonate filters with 0.1 µm pores. Peptides (500 nM) were incubated with 200 µg/ml liposomes in phosphate buffered saline at 37°C for 22 h. Tryptophan fluorescence at 357 nm was measured from at least triplicate trials in the PerkinElmer Life Sciences LS55 spectrofluorometer and an excitation wavelength of 280 nm for SHP-1 and 290 nm for SHP-2, 10 nm excitation and 9 nm emission slit widths. The distance of tryptophans from the bilayer center (Z_CF_) is calculated from the equation [12]:
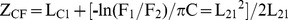
Where L_C1_ is the distance from the center of the bilayer to the shallow quencher, in this case 10.8 Å for 6,7-dibromo-PC [25], F_1_ is the intensity of tryptophan in the presence of the shallow quencher and F_2_ is the tryptophan intensity in the presence of the deep quencher, C is the mole fraction of quencher divided by the area of individual phospholipid (70 Å^2^), and L_21_ is the difference in the depth of the two quenchers (2.7 Å) [25]. The hydrocarbon insertion depth of tryptophans is then given by one half the bilayer thickness, 29 Å [25], minus Z_CF_.

### Circular Dichroism

Spectra were recorded with a Jasco J-710 spectropolarimeter in a 1 mm quartz cuvette. Measurements were performed with a final concentration of 15 µM SHP-1 added from a stock of ethanol-solubilized peptide (final ethanol concentration 7.5 %) in 10 mM K_2_PO_4_, 50 mM Na_2_PO_4_, pH 7.5. Lipid associated peptide spectra were recorded with the addition of 0.1 µm unilamellar egg phosphatidylcholine liposomes at a peptide to lipid ration of 0.3. Spectra were averaged from four scans and the appropriate buffer scans were subtracted.

### Molecular Modeling

The tertiary structure of SHP-1 in a lipid environment was predicted using the web-based molecular dynamics simulation software PEPstr (http://www.imtech.res.in/raghava/pepstr/) [26]. The per-atom charge and radius were calculated by converting the PDB file obtained from PEPstr into a PQR file via the web-based PDB2PQR server (http://kryptonite.nbcr.net/pdb2pqr/) [27]. Values were calculated using the PARSE forcefield. The peptide was subsequently visualized with the PyMOL Molecular Graphics System, Version 1.4 (Schrödinger, LLC).

## Supporting Information

Figure S1 **SHP binding to BSF** ***T. b. brucei*** **.** FITC-labeled SHP-1 (blue – ) and SHP-3 (green – ) were assayed for binding to BSF *T. b. brucei* via flow cytometry (no peptide, red – ) . Trypanosomes were adjusted to 3×10^6^ cells/ml in HMI 9 media with 10 % fetal bovine serum, 8 µM FITC-SHP-1 or FITC-SHP-3 was added and 50,000 cells were immediately counted.
(DOC)

Figure S2 **Trypanosome killing and membrane interaction with SHP tryptophan variants.** **(a)** Small hydrophobic peptide-1 tryptophan variants (Table S1) SHP-1ΔW1 (orange ⧫), SHP-1ΔW8 (green ▪) and SHP-1ΔW18 (red ▴) were tested for trypanocidal activity. **(b)** The ability of SHP-1 tryptophan variants, 1 µM SHP-1ΔW1 (orange – ), 1 µM SHP-1ΔW8 (green – ), 0.2 µM SHP-1 (blue – ) and 0.2 µM SHP-1ΔW18 (red – ), and **(c)** SHP-3 tryptophan variants, 1 µM SHP-3ΔW1 (orange – ), 1 µM SHP-3 (blue – ), 1 µM SHP-3ΔW13 (red – ) and 4 µM SHP-3ΔW20 (green – ), to interact with lipid bilayers was determined by monitoring the release of entrapped calcein from unilamellar egg phosphatidylcholine liposomes.
(DOC)

Table S1 **Sequences and Quenching Data of SHP Tryptophan Variants.**
(DOC)

Methods File S1 **Contains methods for supplementary Figures S1 and S2.**
(DOC)

Movie S1 **Live, untreated BSF** ***T. b. brucei*** **exhibiting normal motility and visualized with DCI video microscopy.**
(MOV)

Movie S2 **Live BSF** ***T. b. brucei*** **visualized via DIC video microscopy approximately 30 sec after addition of 40 µM SHP-3.** Cells exhibit hyperactivated motility.
(MOV)

Movie S3 **Live BSF** ***T. b. brucei*** **visualized via DIC video microscopy approximately 30 sec after addition of 40 µM SHP-3ΔR.** Cells exhibit hyperactivated motility.
(MOV)

Movie S4 **Live BSF** ***T. b. brucei*** **visualized via DIC video microscopy approximately 10 min after addition of 40 µM SHP-3ΔR.** Cells exhibit constricted motility.
(MOV)
