# Supplementary material for: Novel African Trypanocidal Agents: Membrane Rigidifying Peptides
Source: PLoS One. 2012 Sep 7;7(9):e44384. doi: 10.1371/journal.pone.0044384 (PMC3436892; doi:10.1371/journal.pone.0044384)
Supplement: Figure S1 — SHP binding to BSF T. b. brucei . FITC-labeled SHP-1 (blue – ) and SHP-3 (green – ) were assayed for binding to BSF T. b. brucei via flow cytometry (no peptide, red – ) . Trypanosomes were adjusted to 3×10^6^ cells/ml in HMI 9 media with 10 % fetal bovine serum, 8 µM FITC-SHP-1 or FITC-SHP-3 was added and 50,000 cells were immediately counted. (DOC) [file pone.0044384.s001.doc]

**Figure S1.**

**
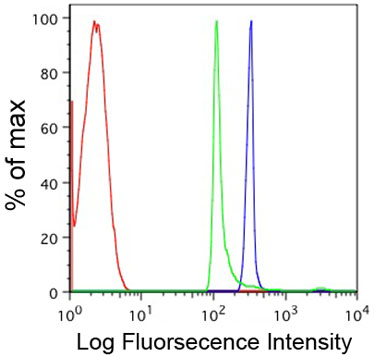
**

**SHP binding to BSF T. b. brucei.** FITC-labeled SHP-1 (blue  ) and SHP-3 (green  ) were assayed for binding to BSF *T. b. brucei* via flow cytometry (no peptide, red  ) . Trypanosomes were adjusted to 3 × 10^6^ cells/ml in HMI 9 media with 10 % fetal bovine serum, 8 μM FITC-SHP-1 or FITC-SHP-3 was added and 50,000 cells were immediately counted.
