# Supplementary material for: Novel African Trypanocidal Agents: Membrane Rigidifying Peptides
Source: PLoS One. 2012 Sep 7;7(9):e44384. doi: 10.1371/journal.pone.0044384 (PMC3436892; doi:10.1371/journal.pone.0044384)
Supplement: Figure S2 — Trypanosome killing and membrane interaction with SHP tryptophan variants. (a) Small hydrophobic peptide-1 tryptophan variants (Table S1) SHP-1ΔW1 (orange ⧫), SHP-1ΔW8 (green ▪) and SHP-1ΔW18 (red ▴) were tested for trypanocidal activity. (b) The ability of SHP-1 tryptophan variants, 1 µM SHP-1ΔW1 (orange – ), 1 µM SHP-1ΔW8 (green – ), 0.2 µM SHP-1 (blue – ) and 0.2 µM SHP-1ΔW18 (red – ), and (c) SHP-3 tryptophan variants, 1 µM SHP-3ΔW1 (orange – ), 1 µM SHP-3 (blue – ), 1 µM SHP-3ΔW13 (red – ) and 4 µM SHP-3ΔW20 (green – ), to interact with lipid bilayers was determined by monitoring the release of entrapped calcein from unilamellar egg phosphatidylcholine liposomes. (DOC) [file pone.0044384.s002.doc]

**Figure S2.**


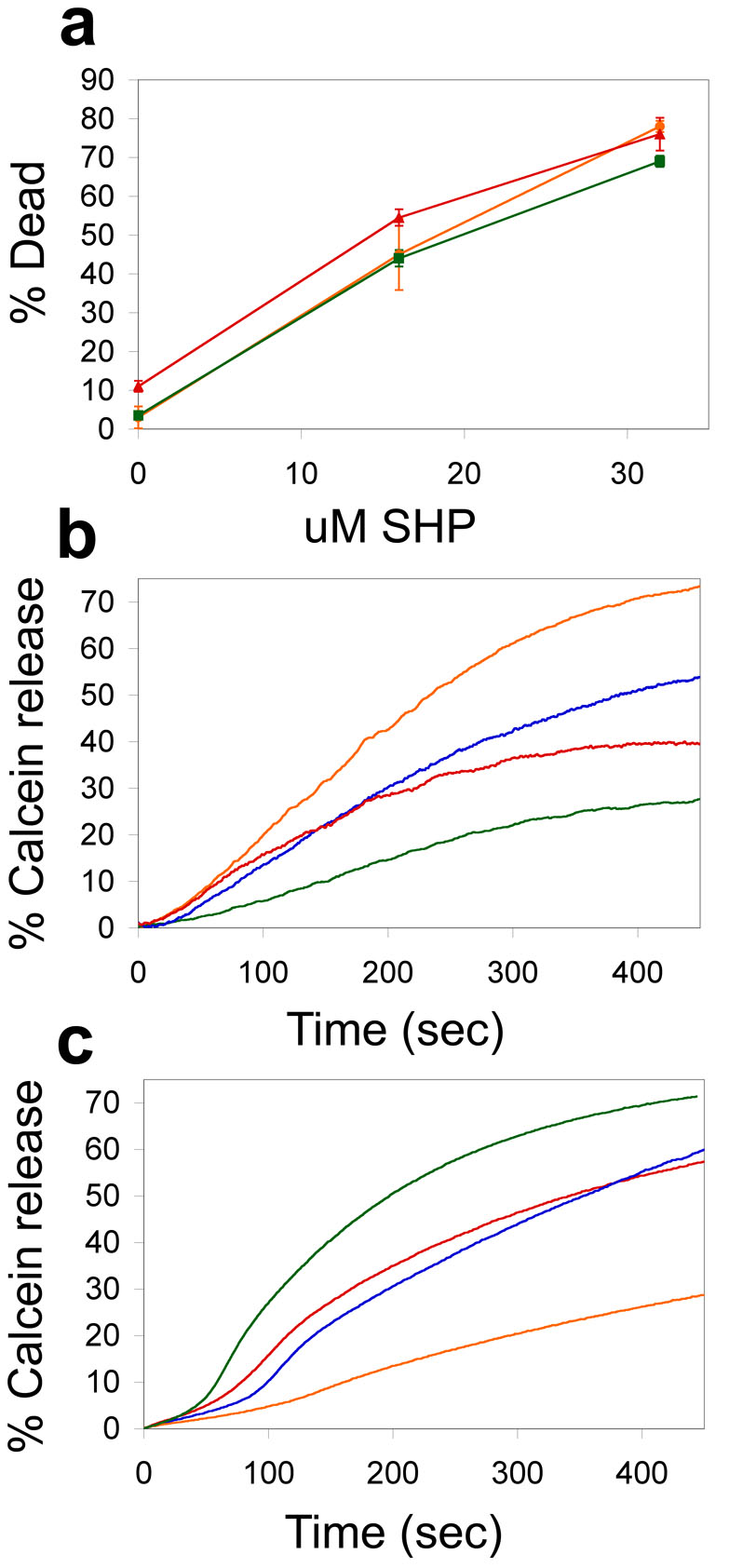


**Trypanosome killing and membrane interaction with SHP tryptophan variants.** **(a)** Small hydrophobic peptide-1 tryptophan variants (Table S1) SHP-1ΔW1 (orange ⬥), SHP-1ΔW8 (green ■) and SHP-1ΔW18 (red ▲) were tested for trypanocidal activity.  **(b)** The ability of SHP-1 tryptophan variants, 1 μM SHP-1ΔW1 (orange  ), 1 μM SHP-1ΔW8 (green  ), 0.2 μM SHP-1 (blue  ) and 0.2 μM SHP-1ΔW18 (red  ), and **(c)** SHP-3 tryptophan variants, 1 μM SHP-3ΔW1 (orange  ), 1 μM SHP-3 (blue  ), 1 μM SHP-3ΔW13 (red  ) and 4 μM SHP-3ΔW20 (green  ), to interact with lipid bilayers was determined by monitoring the release of entrapped calcein from unilamellar egg phosphatidylcholine liposomes.
