# Supplementary material for: Novel African Trypanocidal Agents: Membrane Rigidifying Peptides
Source: PLoS One. 2012 Sep 7;7(9):e44384. doi: 10.1371/journal.pone.0044384 (PMC3436892; doi:10.1371/journal.pone.0044384)
Supplement: Table S1 — Sequences and Quenching Data of SHP Tryptophan Variants. (DOC) [file pone.0044384.s003.doc]

**Table S1. Sequences and Quenching Data of SHP Tryptophan Variants.**

| **Peptide** | **Sequence (N- to C-terminus)^1^** | **F_1_/F_2_** ± S.D.^2^ |
| --- | --- | --- |
| SHP-1∆W1 | WDLGAVISLLLGGRQLFA | 0.8897 ± 0.1112 |
| SHP-1∆W8 | SDLGAVIWLLLGGRQLFA | 0.9767 ± 0.0717 |
| SHP-1 | SDLGAVISLLLWGRQLFA | 0.9676 ± 0.0560 |
| SHP-1∆W18 | SDLGAVISLLLGGRQLFW | 0.9285 ± 0.1557 |
| SHP-3∆W1 | WHQIGAALLYFYGIILNSIY | N.A. |
| SHP-3 | FHQIWAALLYFYGIILNSIY | 0.9202 ± 0.1260 |
| SHP-3∆W13 | FHQIGAALLYFYWIILNSIY | 0.9912 ± 0.0787 |
| SHP-3∆W20 | FHQIGAALLYFYGIILNSIW | 1.1620 ± 0.3194 |

^1^Peptide sequences illustrate the different positions of tryptophan substitutions (in red).

^2^Quenching data is presented as the ratio of tryptophan fluorescence intensity in the presence of the shallow, F_1_, and deep, F_2_, quencher and the standard deviations (S.D.).
