# Supplementary material for: Novel African Trypanocidal Agents: Membrane Rigidifying Peptides
Source: PLoS One. 2012 Sep 7;7(9):e44384. doi: 10.1371/journal.pone.0044384 (PMC3436892; doi:10.1371/journal.pone.0044384)
Supplement: Methods File S1 — Contains methods for supplementary Figures S1 and S2. (DOC) [file pone.0044384.s004.doc]

**Supplementary Material for**

**Novel African Trypanocidal Agents: Membrane Rigidifying Peptides**

**John M. Harrington^1^, Chris Scelsi^1^, Andreas Hartel^2^, Nicola G. Jones^2^, Markus Engstler^2^, Paul Capewell^3^, Annette MacLeod^3^ and Stephen Hajduk^1^***

^1^Department of Biochemistry and Molecular Biology, University of Georgia, Athens GA 30602

^2^Department of Cell and Developmental Biology, Theodor-Boveri-Institute, University of Wuerzburg, Am Hubland, 97074 Wuerzburg, Germany

^3^Wellcome Trust Centre for Molecular Parasitology, College of Medical, Veterinary and Life Sciences, University of Glasgow, 464 Bearsdon Road, Glasgow, G61 1QH, UK

*e-mail: [shajduk@bmb.uga.edu](mailto:shajduk@bmb.uga.edu)

**Supplementary Methods**

Flow Cytometry – Peptide binding to BSF *T. b. brucei* was monitored by flow cytometry as described previously [1]. Binding assays were performed with 3 × 10^6^ cells/ml in HMI 9 plus 10 % fetal bovine serum at 25 °C. FITC-labeled SHP-1 or SHP-3 was added to a final concentration of 8 μM and 50,000 cells were immediately counted on a CyAn ADP flow cytometer (Dako). Data were analyzed with FlowJo software (TreeStar Inc.).

Calcein Release Assays – Membrane permeabilization assays were conducted as described in detail previously [1, 2]. Unilamellar liposomes were constructed as described above but with 30 mM calcein in 10 mM Hepes as the hydration buffer. Untrapped dye was removed by gel filtration (Sephacryl S-300 HR, GE Healthcare). Liposomes were diluted 1:1000 into phosphate buffered saline, and calcein fluorescence was monitored at 513 nm when excited at 484 nm. The percent calcein release was calculated relative to the 100% fluorescence intensity, achieved by the addition of 0.01% Triton X-100.

**Supplementary References**

1. Harrington JM, Widener J, Stephens N, Johnson T, Francis M et al. (2010) The plasma membrane of bloodstream-form African trypanosomes confers susceptibility and specificity to killing by hydrophobic peptides. J Biol Chem 285: 28659-28666.

2. Harrington JM, Howell S, Hajduk SL (2009) Membrane permeabilization by trypanosome lytic factor, a cytolytic human high-density lipoprotein. J Biol Chem 284: 13505-13512.
